# Website Use and Effects of Online Information About Tobacco Additives Among the Dutch General Population: A Randomized Controlled Trial

**DOI:** 10.2196/jmir.6785

**Published:** 2017-03-14

**Authors:** Dominique A Reinwand, Rik Crutzen, Anne S Kienhuis, Reinskje Talhout, Hein de Vries

**Affiliations:** ^1^ Rehabilitative Gerontology Faculty of Human Science University of Cologne Cologne Germany; ^2^ CAPHRI Department of Health Promotion Maastricht University Maastricht Netherlands; ^3^ Center for Health Protection National Institute for Public Health and the Environment Bilthoven Netherlands

**Keywords:** tobacco additives, information dissemination, website use, website evaluation, RCT

## Abstract

**Background:**

As a legal obligation, the Dutch government publishes online information about tobacco additives to make sure that it is publicly available. Little is known about the influence this website (”tabakinfo”) has on visitors and how the website is evaluated by them.

**Objective:**

This study assesses how visitors use the website and its effect on their knowledge, risk perception, attitude, and smoking behavior. The study will also assess how the website is evaluated by visitors using a sample of the Dutch general population, including smokers and nonsmokers.

**Methods:**

A randomized controlled trial was conducted, recruiting participants from an online panel. At baseline, participants (N=672) were asked to fill out an online questionnaire about tobacco additives. Next, participants were randomly allocated to either one of two experimental groups and invited to visit the website providing information about tobacco additives (either with or without a database containing product-specific information) or to a control group that had no access to the website. After 3 months, follow-up measurements took place.

**Results:**

At follow-up (n=492), no statistically significant differences were found for knowledge, risk perception, attitude, or smoking behavior between the intervention and control groups. Website visits were positively related to younger participants (B=–0.07, 95% CI –0.12 to –0.01; t_11_=–2.43, *P*=.02) and having a low risk perception toward tobacco additives (B=–0.32, 95% CI –0.63 to –0.02; t_11_=–2.07, *P*=.04). In comparison, having a lower education (B=–0.67, 95% CI –1.14 to –0.17; t_11_=–2.65, *P*=.01) was a significant predictor for making less use of the website. Furthermore, the website was evaluated less positively by smokers compared to nonsmokers (t_324_=–3.55, *P*<.001), and males compared to females (t_324_=–2.21, *P*=.02).

**Conclusions:**

The website did not change perceptions of tobacco additives or smoking behavior. Further research is necessary to find out how online information can be used to effectively communication about the risks of tobacco additives.

**Trial Registration:**

Nederlands Trial Register NTR4620; http://www.trialregister.nl/trialreg/admin/rctview.asp?TC=4620 (Archived by WebCite at http://www.webcitation.org/6oW7w4Gnj)

## Introduction

Cigarettes and other manufactured tobacco products contain numerous additives [1]. A total of 673 different tobacco additives are used during the production of cigarettes [2]. Indeed, one cigarette contains, on average, 68 different additives [3]. Additives such as sugar or vanillin may be perceived as being harmless, but they can develop into harmful carcinogenic substances (eg, formaldehyde) during the combustion process [1,4,5]. Some tobacco additives are thought to cause a higher bioavailability of nicotine, which increases nicotine addiction [6]. From the perspective of those in the tobacco industry, one of the main reasons to use tobacco additives is to improve the taste and to make the smoke milder and consumable [6].

Since 2003, tobacco producers and importers in the Netherlands are legally obliged to list all tobacco additives used in their products and to provide this information to the government [7]. Information about the amount of additives used—including their function and known impact on health—must be declared [8]. Since 2012, the Dutch National Institute for Public Health and the Environment (RIVM) has published information about tobacco additives on a dedicated website (“tabakinfo”). The aim of this website is to provide neutral and objective information to the general population. In contrast to the Netherlands, most other European countries that collect information about tobacco additives do not publish this information. In the United Kingdom, for example, this information is not published because it is feared that people might misunderstand the information [3].

Only two studies—one in Australia [9] and one in the United States [10]—have investigated the influence of the public dissemination of information about components in tobacco or smoke. Results from focus group interviews in Australia demonstrated that information about tobacco additives is desired by the general public. In this case, despite feeling that information about tobacco additives might have an influence on smoking behavior, they were not interested in looking up this information. Moreover, some information appeared to be too detailed or confusing. Additionally, information related to tobacco additives led to some misconceptions. For example, some people thought that cigarettes with fewer additives were less dangerous than cigarettes containing more additives [9].

A cross-sectional quantitative survey in the United States looked at the impact of information about smoke components on smokers and nonsmokers by assessing awareness, worries, and smoking discouragement [10]. The study participants were aware of six of the 20 mentioned components. Reading information related to tobacco additives was associated with increased levels of worry about the harmfulness of substances that were added to tobacco products, such as tobacco additives, compared to substances that naturally occur in cigarette smoke. However, smoke components differ from tobacco additives. Tobacco additives are intentionally added during the manufacturing process of the tobacco product to improve taste or product quality, whereas smoke components are substances to which smokers are exposed to during use of the product. Still, the US study is informative with regard to how people perceive information on tobacco additives.

It is conceivable that information about tobacco additives and smoke components may not be interesting to everyone. Smokers are known to be less interested in information about the risks of smoking [11] in comparison to nonsmokers. It might also be that people with different educational levels understand information about tobacco additives and smoke components differently, or might even misunderstand them. Previous studies that underpin this assumption are rarely about tobacco additives, but have been conducted for potential reduced exposure products (PREPs) (eg, light cigarettes). Some studies demonstrated that smokers believed that light products were less harmful compared to regular cigarettes [12,13]. Although the information given about PREPs in these studies did not contain any statements about positive health outcomes, smokers thought that these products were healthier compared to regular cigarettes [12]. Furthermore, those with a higher education level were more aware of those products. However, no differences were found with regard to risk perceptions of these products when comparing different educational levels [14].

Another study about reactions to reduced risk tobacco advertisements found that people with a lower educational level misinterpreted the advertisement and thought those products were completely free of health risks [15]. Moreover, those with a higher educational level used PREPs more frequently than those with a lower educational level [16]. These results show that information can have a misleading impact. There is a risk that smokers and nonsmokers, as well as people with different educational levels, can misunderstand information provided online about tobacco additives and smoke components. Given that information on tobacco additives is mandatory nowadays, it is important to find out whether this information may result in changing knowledge, risk perceptions, and attitudes about tobacco additives. Indeed, these determinants are important in the intention-forming process in health-related behaviors [17-19]. Currently, there is a lack of insight into these determinants in the Netherlands.

The current website has the aim to provide information to the general public, not to change attitudes and behavior, a goal also strictly guided by policies from the Dutch government on these matters. Yet, because the website could influence attitudes and behavior, these outcomes were also the subject of our evaluation, as well as assessing potential negative side effects of the new website on these outcomes. Therefore, the first aim of our study is to describe the effects of a randomized controlled trial (RCT) in terms of knowledge, risk perception, attitude change about tobacco additives, and smoking behavior. This will be in terms of the amount of daily smoked cigarettes. The second aim is to assess the usage of the website. Finally, we will describe evaluation of the website by the visitors.

## Methods

### The Tabakinfo Website

For this study, we made a copy of the original website and deactivated hyperlinks to information that was not about tobacco additives, which were also available on the original website. By doing this, we wanted to reduce attention to other information and be able to measure the usage behavior of participants. On the tabakinfo website, participants could find information about legislation on tobacco additives, why tobacco additives are used, and in which products they are added. Furthermore, 15 specific tobacco additives (eg, sugar or vanillin) were listed on fact sheets. These provided more information about the function of these additives and their potential harms. The website also provided a database where visitors could search for specific products by brand and get an overview about composition and additives.

### Design

**Figure 1:**
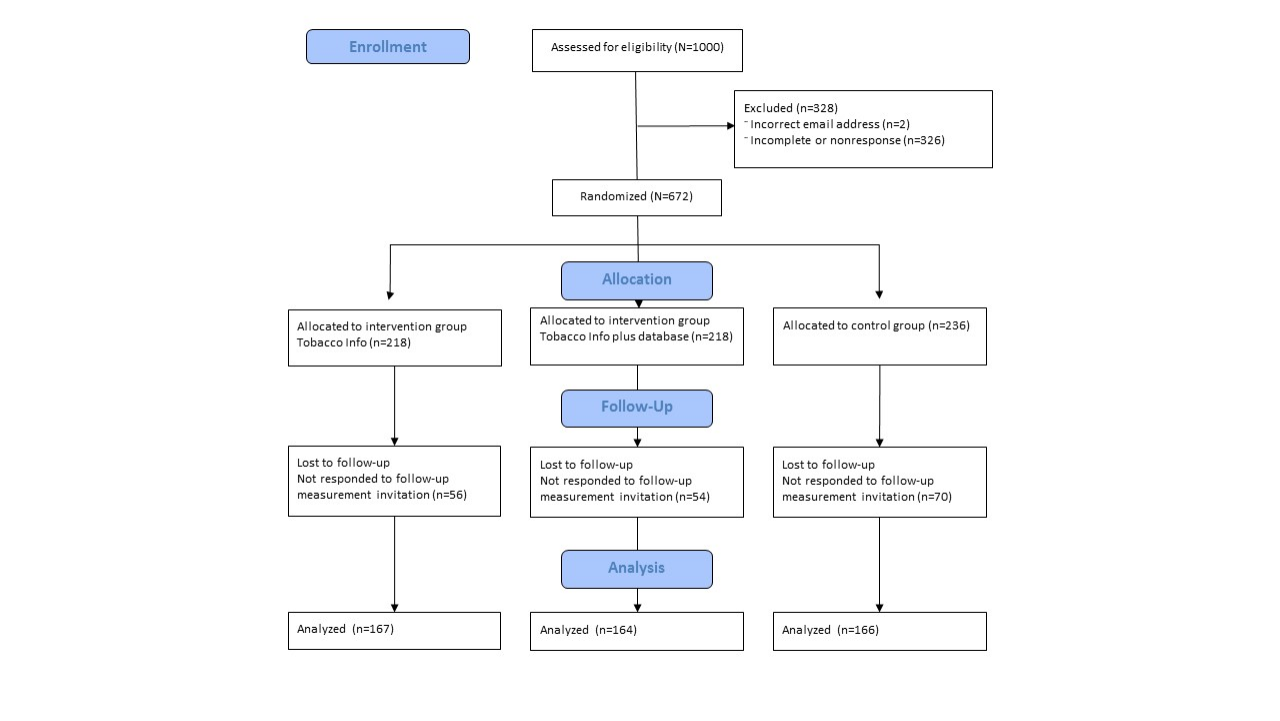
Flowchart of study design.

An RCT with three study groups (tobacco info, tobacco info plus database, and control group) and two measurement times (baseline and follow-up) was conducted (Figure 1). Participants in all three groups had to fill in the baseline questionnaire. Afterwards, the tobacco info group was asked to visit the website and to read information about tobacco additives. The tobacco info plus database group was asked to visit the website and also the database. They looked up information about tobacco additives for a cigarette brand and type. This information was not available on the website for participants who were allocated to the tobacco info group. This manipulation was done in order to investigate the possible effect of visiting the database. The follow-up measurement took place after 3 months. During the 3 month, participants had the opportunity to visit the original website. The control group only had to complete the baseline and follow-up questionnaires but did not have access to the website. Ethical approval of the Regional Medical Ethics committee in the Netherlands was not necessary because participants in this study were not subjected to procedures or required to follow certain rules of behavior (the criteria for ethical approval) [20].

### Procedure and Participants

Participants older than 18 years were recruited via a Dutch independent Internet panel that operates in line with ISO standards [21]. All members of this panel expressed their willingness to participate in scientific research studies. In total, 1000 panel members were invited to participate in this study, of which 672 panel members did (67.21% response rate). Data collection took place in July (baseline) and October (3-month follow-up) 2014. After receiving an email invitation, participants had 1 week to fill in the questionnaire. One email reminder was sent to participants who had not responded after 5 days. To be able to make a comparison between smoker and nonsmoker perceptions of the website, we aimed to have 50% of the participants in the study sample as smokers and 50% as nonsmokers. Panel members received an email with a link to the online questionnaire. At the end of the questionnaire, a unique link to the study website was included for participants from one of the experimental groups. The email for participants allocated to the control group did not include this. The use of a unique link for each participant from the experimental groups provided the opportunity to track behavior of the participants on the website (ie, what parts of the website the participants visited) using the software ComScore by Sitestat [22]. The invitation for the follow-up measurement was only sent to participants who completed the baseline questionnaire. Participants received incentives in the form of points they can collect and exchange for vouchers.

### Questionnaire

The questionnaire was administered in Dutch. Personal characteristics were assessed only at baseline and included gender (1=male, 2=female), age (1=18-19, 2=20-24, 3=25-29, 4=30-34, 5=35-39, 6=40-44, 7=45-49, 8=50-54, 9=55-59, 10=60-64, 11=65 years or older), educational status (1=low: no education, primary or lower vocational school, 2=middle: secondary vocational school or high school, 3=high: higher education or university), and income (1=low: <€12,500; 2=middle: €26,000-€39,000; 3=high: €39,000 or more). As described by Statistics Netherlands, distribution of the sample was representative of the Dutch population with regard to age, gender, income, and level of education [23].

At baseline, participants were asked with one item if they already knew the website: “Are you familiar with the tabakinfo website?” (1=yes, 2=no).

Smoking behavior, knowledge, risk perception, and attitude toward tobacco additives were measured at baseline and at 3-month follow-up. In line with World Health Organization guidelines for measurements of smoking status, all participants were asked whether they smoked at least once a month (1=yes, 0=no) [24]. If yes, participants were categorized as smokers. They were then asked to indicate through multiple choice questions how many they smoked a day, the brand they smoked, and the type of cigarettes they smoked (eg, cigarillos, cigars, rolling tobacco, or pipe).

Omega was used to assess the internal structure of the scales [25]; this value represents a less biased alternative to Cronbach alpha [26]. Similar to Cronbach alpha, omega values can range from 0 to 1, where higher values indicate a more coherent internal structure. In other words, the proportion of variance due to a general factor (ie, omega) provides important information about the extent to which a scale score estimates a latent variable common to all items [27]. Knowledge about tobacco additives was assessed with 10 right or wrong statements (omega=0.82, 95% CI 0.80-0.84). To dichotomize knowledge, we recoded “I don’t know” answers as answering the question wrong because they did not know the right answer. For example, “Tobacco with additives consists of less carcinogenic substances compared to tobacco without additives” (wrong).

To measure risk perception toward tobacco additives, six items were assessed; three items covered cognitive aspects and three items covered affective aspects of risk perception. These questions were based on earlier questionnaires assessing risk perception and were adjusted for tobacco additives [28]. Answers were given on a five-point Likert scale (5=totally agree, 1=totally disagree; omega=0.81, 95% CI 0.77-0.84). “If I smoke tobacco with additives I have a high chance of getting cancer,” is an example item of a cognitive risk perception item.

Attitude toward tobacco additives was assessed by 14 items on a five-point Likert scale (5=totally agree, 1=totally disagree); there were eight items about the pros with regard to tobacco additives and six items about the cons of tobacco additives (pro: omega=0.83, 95% CI 0.79-0.86; con: omega=0.79, 95% CI 0.76-0.82). These questions were based on an earlier questionnaire assessing attitude and were adjusted for tobacco additives [29,30]. As an example, for a pro attitude item, participants were asked, “If I would smoke tobacco with additives, I feel good.”

Items with regard to the evaluation of the questionnaire was included in all studies that were conducted in the panel used in the study at hand. On a visual analog five-point Likert scale, participants rated whether they evaluated the questionnaire as interesting (1) or uninteresting (5), pleasurable (1) or not (5), too long (5) or too short (1), and difficult (1) or easy (5) to answer. We also documented how many minutes it took to complete the questionnaire.

The follow-up measurement contained items about the website evaluation, which were only answered by participants from the two experimental groups. Participants’ perceptions about the website were assessed using 10 concepts about diverse aspects of the website, such as content, layout, language, or navigation [31]. These different constructs are useful in evaluating different aspects of visitor experiences on the website. Positive experiences are associated with detailed use [32]. Completeness and layout were assessed with two items, and the other concepts with three items each: efficiency (eg, I easily find information I am looking for on this website; omega=0.92, 95% CI 0.90-0.93), effectiveness (eg, The website provides useful information; omega=0.92, 95% CI 0.91-0.94), enjoyment (eg, I found my visit on this website enjoyable; omega=0.96, 95% CI 0.95-0.96), active trust (eg, I would act on the information presented on this website if needed; omega=0.89, 95% CI 0.86-0.91) [31,33], relevance (eg, The information on the website was new to me; omega=0.78, 95% CI 0.71-0.80), understanding (eg, I found many words on the website difficult to understand; omega=0.83, 95% CI 0.82-0.88), completeness (eg, The website contains enough information; *r*=.79), layout (eg, I found that the layout of the website looks good; *r*=.29), recommendation to others (eg, I would recommend the website to others; omega=0.83, 95% CI 0.84-0.89), and intention to revisit (eg, I would revisit the website again; omega=0.84, 95% CI 0.81-0.87). All items could be answered on a five-point Likert scale (1=totally disagree, 3=neither disagree nor agree, 5=totally agree).

Website use was assessed based on tracking the unique links that participants received. Using these unique links, it was possible to see which pages each participant visited.

### Statistical Analyses

Data was analyzed using the software SPSS version 24 and R was used to calculate omegas as scale reliability for knowledge, risk perception, attitude pro, attitude con, and website evaluation constructs. Logistic regression analysis was used to evaluate whether dropout was higher among specific subgroups. Frequencies and descriptive statistics were used to describe the study sample at baseline.

Linear regression using the enter method was used to analyze differences in knowledge, risk perception, attitude, and the amount of cigarettes smoked per day between control and study groups after visiting the website. All analysis was done for these five outcome variables, with age, gender, level of education and income, and smoking status as independent variables. Further, interaction between of study group*age, study group*smoking status, and study group*education were used in order to determine any differences among the subgroups using the website. If one of these interaction terms were significant at a *P* value of ≤.05, analyses on the relevant subgroups were undertaken. All analyses were corrected for age, gender, educational level, income, and smoking status. When analyzing the follow-up effects of knowledge, risk perception, attitude pro, attitude con, and smoking, the corresponding baseline measurement was included on each of the analysis.

Linear regression analyses using the enter method were conducted to determine the predictors of website use. For this purpose, a continuous dependent variable was calculated which summed up the numbers of visited pages within the website. This included specific information about tobacco additives. Independent variables included age, gender, education, income, knowledge, attitude, risk perception, smoking status, amount of smoked cigarettes, duration of questionnaire, and questionnaire evaluation. In total, 19 participants reported at baseline that they knew the website (tobacco info group: n=9; tobacco info plus database group: n=7; control group: n=3) and were excluded from analyses.

Finally, we analyzed how the website was evaluated by the different subgroups. For this purpose, we conducted independent sample *t* tests to compare males with females, and smokers with nonsmokers, using ANOVA Tukey post hoc tests we compared educational and income levels and used the means of each of the 10 evaluation concepts (efficiency, effectiveness, active trust, relevance, enjoyment, understanding, completeness, layout, recommendation to others, and intention to revisit).

## Results

### Sample Characteristics and Attrition

The study sample (Table 1) consisted of 672 participants (tobacco info plus database group: n=218; tobacco info group: n=218; control group: n=236) at baseline (male: 54.8%, 368/672). In terms of age and gender, the distribution within the study sample was in line with distribution in the Netherlands, as was distribution of income (high: 31.7%, 213/672; middle: 45.2%, 304/672; low: 23.1%, 155/672) and education (high: 24.6%, 165/672; middle: 43.2%, 290/672; low: 32.3%, 217/672). Furthermore, the sample consisted of 53.9% (632/672) smokers. Table 2 presents perceptions about tobacco additives at baseline and follow-up for each of the study groups.

**Table 1:** Participant characteristics (N=672).

Variables		n (%)
**Age (years)**		
	18-19	23 (3.4)
	20-24	58 (8.9)
	25-29	61 (9.1)
	30-34	50 (7.4)
	35-39	47 (7.0)
	40-44	60 (8.9)
	45-49	69 (10.3)
	50-54	66 (9.8)
	55-59	61 (9.1)
	60-64	50 (7.4)
	≥65	127 (18.9)
**Gender**		
	Male	368 (54.8)
	Female	304 (45.2)
**Smoking**		
	Smoker	362 (53.9)
	Nonsmoker	310 (46.1)
**Education**		
	Low	217 (32.3)
	Middle	290 (43.2)
	High	165 (24.6)
**Income**		
	Low	155 (23.1)
	Middle	304 (45.2)
	High	213 (31.7)

**Table 2:** Baseline and 3-month follow-up perceptions about tobacco additives.

Variables		Tobacco info group, mean (SD)	Tobacco info plus database group, mean (SD)	Control group, mean (SD)
**Knowledge (score 0-1)**				
	Baseline	0.42 (0.28)	0.44 (0.28)	0.42 (0.29)
	Follow-up	0.56 (0.28)	0.54 (0.28)	0.53 (0.26)
**Risk perception (score 1-5)**				
	Baseline	3.45 (0.62)	3.42 (0.61)	3.41 (0.61)
	Follow-up	3.47 (0.61)	3.49 (0.60)	3.45 (0.66)
**Attitude pro (score 1-5)**				
	Baseline	2.64 (0.52)	2.54 (0.62)	2.60 (0.64)
	Follow-up	2.53 (0.58)	2.53 (0.60)	2.43 (0.63)
**Attitude con (score 1-5)**				
	Baseline	3.40 (0.58)	3.50 (0.64)	3.43 (0.67)
	Follow-up	3.48 (0.66)	3.49 (0.61)	3.55 (0 73)

The loss to follow-up after 3 months was 26.8% (180/672). Dropout was significantly higher for participants with a lower income (OR 0.42, 95% CI 0.19-0.90, *P*=.03), higher within the experimental group tobacco info plus database (OR 2.17, 95% CI 1.12-4.22, *P*=.02), and among participants who indicated at baseline that they did not like filling out the questionnaire (OR 0.48, 95% CI 0.30-0.77, *P*=.002). With regard to all other variables, including age, gender, smoking behavior, educational level, evaluation of the questionnaire (clear: mean 1.9, SD 1.1; interesting: mean 2.1, SD 1.2; length: mean 3.0, SD 0.6; pleasurable: mean 2.1, SD 1.1), for knowledge, attitude, and risk perception, no significant differences in dropout were found.

### Determinants and Smoking Status

Table 2 indicates the mean values of tobacco additives perceptions at baseline and follow-up. ANOVA and Tukey post hoc tests did not reveal differences between the three study groups. As can been seen in Tables 3 and 4, after the 3-month follow-up, when compared to the control group, neither one of the measured concepts with regard to tobacco additives (knowledge: *R*^2^=.39; risk perception: *R*^2^=.30; attitude pro: *R*^2^=.34; attitude con: *R*^2^=.38) or the amount of cigarettes smoked per day (*R*^2^=.62) changed within the experimental groups. Furthermore, smokers had significantly lower risk perception and a less negative attitude toward tobacco additives than nonsmokers. None of the interaction terms were significant, indicating that there were no subgroup effects for smoking status, people with different educational levels, and age groups.

**Table 3:** Linear regression results for knowledge, risk perception, and attitude pro after follow-up measurement among study groups.

Variables	Knowledge		Risk perception		Attitude pro	
	B (95% CI)^a^	*P*	B (95% CI)^a^	*P*	B (95% CI)^a^	*P*
Tobacco info group	0.08 (–0.14, 0.30)	.46	0.22 (–0.31, 0.75)	.42	–0.11 (–0.61, 0.39)	.67
Tobacco info + database group	0.16 (–0.07, 0.38)	.17	0.52 (–0.02, 1.07)	.06	–0.27 (–0.78, 0.24)	.30
Age	0.00 (–0.01, 0.01)	.77	0.02 (–0.01, 0.05)	.24	0.01 (–0.02, 0.03)	.70
Gender	–0.01 (–0.06, 0.03)	.52	–0.03 (–0.14, 0.07)	.52	–0.04 (–0.13, 0.06)	.48
Education low	–0.03 (–0.12, 0.07)	.61	–0.17 (–0.41, 0.06)	.14	0.05 (–0.17, 0.27)	.68
Education middle	–0.05 (–0.14, 0.03)	.21	–0.10 (–0.30, 0.10)	.34	–0.06 (–0.25, 013)	.54
Income low	0.01 (–0.04, 0.07)	.65	–0.10 (–0.23, 0.04)	.16	0.05 (–0.08, 0.17)	.48
Income middle	0.00 (–0.05, 0.04)	.93	0.10 (–0.01, 0.21)	.08	0.02 (–0.09, 0.12)	.76
Smoking status	0.05 (–0.02, 0.12)	.17	0.19 (0.02, 0.36)	.02	–0.11 (–0.27, 0.05)	.19
Tobacco info group*age	0.00 (–0.02, 0.02)	.96	–0.02 (–0.06, 0.02)	.27	0.01 (–0.03, 0.05)	.60
Tobacco info + database group*age	–0.01 (–0.03, 0.01)	.26	–0.02 (–0.06, 0.02)	.23	0.00 (–0.04, 0.04)	.90
Tobacco info group*education low	0.01 (–0.12, 0.14)	.88	0.06 (–0.26, 0.38)	.71	–0.05 (–0.35, 0.26)	.77
Tobacco info group*education middle	0.05 (–0.07, 0.17)	.43	0.09 (–0.19, 0.38)	.52	0.00 (–0.27, 0.28)	>.99
Tobacco info + database group*education low	–0.02 (–0.16, 0.11)	.76	0.03 (–0.30, 0.35)	.87	0.25 (–0.06, 0.56)	.11
Tobacco info + database group*education middle	0.01 (–0.11, 0.14)	.82	–0.14 (–0.44, 0.16)	.35	0.12 (–0.17, 0.40)	.42
Tobacco info group*smoking	–0.04 (–0.14, 0.06)	.39	–0.09 (–0.32, 0.15)	.48	0.06 (–0.17, 0.29)	.60
Tobacco info + database group*smoking	–0.04 (–0.14, 0.06)	.42	–0.17 (–0.41, 0.07)	.16	0.16 (–0.06, 0.39)	.16
Baseline assessment^b^	0.60 (0.52, 0.67)	<.001	0.47 (0.39, 0.55)	<.001	0.55 (0.47, 0.63)	<.001

^a^ Unstandardized B.

^b^ Baseline assessment for the corresponding outcome.

**Table 4:** Linear regression results for outcome for attitude con and cigarettes per day after follow-up measurement among study groups.

Variables	Attitude con		Cigarettes per day	
	B (95% CI)^a^	*P*	B (95% CI)^a^	*P*
Tobacco info group	0.12 (–0.42, 0.65)	.66	–0.22 (–0.86, 0.42)	.50
Tobacco info + database group	0.40 (–0.14, 0.95)	.15	–0.23 (–0.86, 0.40)	.48
Age	0.00 (–0.03, 0.03)	.90	0.02 (–0.03, 0.07)	.38
Gender	0.06 (–0.05, 0.16)	.27	0.00 (–0.16, 0.15)	.96
Education low	0.06 (–0.18, 0.29)	.64	0.09 (–0.23, 0.41)	.59
Education middle	–0.13 (–0.34, 0.07)	.21	–0.02 (–0.32, 0.29)	.92
Income low	–0.02 (–0.16, 0.11)	.75	0.04 (–0.15, 0.23)	.66
Income middle	0.06 (–0.06, 0.17)	.32	0.13 (–0.03, 0.30)	.11
Smoking status	0.27 (0.10, 0.45)	<.001	—	
Tobacco info group*age	0.00 (–0.04, 0.04)	.85	0.00 (–0.07, 0.06)	.91
Tobacco info + database group*age	–0.01 (–0.05, 0.03)	.69	–0.01 (–0.07, 0.06)	.87
Tobacco info group*education low	–0.12 (–0.44, 0.20)	.48	0.0.9 (–0.36, 0.55)	.68
Tobacco info group*education middle	0.23 (–0.06, 0.53)	.11	0.32 (–0.13, 0.77)	.16
Tobacco info + database group*education low	–0.34 (–0.67,–0.01)	.40	0.21 (–0.25, 0.68)	.37
Tobacco info + database group*education middle	–0.13 (–0.44, 0.17)	.39	0.19 (–0.28, 0.66)	.42
Tobacco info group*smoking	–0.17 (–0.41, 0.07)	.17	—	
Tobacco info + database group*smoking	–0.15 (–0.39, 0.09)	.23	—	
Baseline assessment^B^	0.54 (0.46, 0.63)	<.001	0.75 (0.67, 0.84)	<.001

^a^ Unstandardized B.

^b^ Baseline assessment for the corresponding outcome.

### Website Use

All participants from the tobacco info group visited the website. From the tobacco info plus database group, 159 of 163 participants (97.6%) visited the website; specific information about tobacco additives, another part of the website, was looked up by 129 of 346 (37.3%) participants from both groups. Only a small minority of participants browsed the website to gain more information about one of the 14 specific tobacco additives, such as vanilla (23/346, 6.7%) or sugar (25/346, 7.2%). Of those participants from the tobacco info plus database group who visited the website (n=159), 33.3% (53/159) visited the subwebsite that provided the database.

The model in Table 5 accounted for 78% of the total variance in predicting website usage. It shows that being younger (B=–0.07, *t*_11_=–2.43, *P*=.03) and having a low risk perception toward tobacco additives (B=–0.32, *t*_11_=–2.07, *P*=.04) were significant predictors for website usage, but having a lower educational level (B=–0.67, *t*_11_=–2.65, *P*=.01) was a significant predictor for using the website less.

**Table 5:** Linear regression analysis of predictors of website use (n=337).

Variables	Website use	
	B (95% CI)^a^	*P*
Smoking	–0.08 (–0.43, 0.27)	.66
Age	–0.07 (–0.12,–0.01)	.02
Gender	–0.15 (–0.48, 0.18)	.37
Education low	–0.66 (–1.14,–0.17)	.05
Education middle	–0.32 (–0.75, 0,11)	.14
Income low	–0.03 (–0.51, 0.44)	.89
Income middle	–0.10 (–0.49, 0.29)	.61
Knowledge	0.43 (–0.21, 1.07)	.19
Risk perception	–0.32 (–0.63,–0.02)	.04
Attitude pro	–0.05 (–0.39, 0.28)	.75
Attitude con	0.10 (–0.25, 0.46)	.57

^a^ Unstandardized B.

### Website Evaluation

The website was evaluated through use of the following 10 concepts: efficiency, effectiveness, active trust, relevance, enjoyment, understanding, completeness, layout, recommendation to others, and intention to revisit. Table 6 shows the mean scores of these scales for the study sample. As can be seen, participants tended to evaluate all these concepts negatively because their answers ranged between disagree and neutral.

**Table 6:** Website evaluation.

Evaluation variables	Tobacco info group, mean (SD) (n=158)	Tobacco info plus database group, mean (SD) (n=157)
Enjoyment	2.73 (0.70)	2.38 (0.65)
Layout	2.58 (0.58)	2.67 (0.63)
Intention to revisit	2.74 (0.77)	2.88 (0.86)
Active trust	2.60 (0.70)	2.61 (0.69)
Recommending to others	2.64 (0.70)	2.66 (0.73)
Relevance	2.38 (0 65)	2.48 (0.59)
Completeness	2.32 (0.58)	2.41 (0.69)
Efficiency	2.28 (0.62)	2.38 (0.65)
Effectiveness	2.25 (0.61)	2.33 (0.64)
Understanding	2.22 (0.66)	2.34 (0.65)

^a^ Scales: 1=totally disagree, 3=neither disagree nor agree, 5=totally agree.

For all these concepts, we did not find significant differences between participants with different educational levels, income groups, or different ages. Females (mean 2.70, SD 0.73) were found to put a significantly higher degree of active trust in the website compared to males (mean 2.53, SD 0.65; mean difference=–0.17, 95% CI –0.33 to –0.17; *t*_324_=–2.21, *P*=.03). Furthermore, smokers (mean 2.66, SD 0.79) compared to nonsmokers (mean 2.90, SD 0.80) had a significantly lower intention to revisit the website again (mean difference=–0.32, 95% CI –0.49 to –0.14; *t*_324_=–3.55, *P<*.001).

## Discussion

### Main Findings

This study showed that participants at baseline did not have a high level of knowledge about tobacco additives, which is in line with a recent study from the United States [34]. Furthermore, our participants had no strong positive or negative risk perception or attitude regarding tobacco additives.

We demonstrated that, at 3-month follow-up, visiting the website resulted in no changes between the control group and the experimental groups. This could be explained by several factors. Firstly, the participants from our study visited the website only once. This single exposure to new information might not be enough to expect any changes in smoking behavior, nor determinants such as knowledge, risk perception, or attitude [35]. Furthermore, participants showed low levels of engagement with the website in terms of visiting subwebsites and specific information. Also, participants did not evaluate the website positively and had low intentions to revisit the website, which could have limited the impact of the website. In addition, it might also be possible that participants from the control group became curious about the topic of tobacco additives and searched for more information about this topic (eg, using Google). Those participants might have visited the original website and received the same information about tobacco additives as the experimental groups or found other information about tobacco additives on the Web.

Secondly, with regard to predictors of website use, we found that personal characteristics, such as an older age, higher educational level, and having a high risk perception toward tobacco additives is associated with more extensive website use. This included visiting more subwebsites (ie, looking up specific information about tobacco additives). This is in line with previous findings that people with a higher socioeconomic status are more interested in health-related topics and search the Internet more frequently for health information [36,37]. Furthermore, a content analysis of information provided online on tobacco indicated that most websites require high grades of reading levels [38]. These requirements might explain why participants with a lower educational level visited less information on the evaluated website. Besides, participants with a low risk perception toward tobacco additives had visited the website in more detail, supporting earlier findings indicating that high risk perception is associated with the avoidance of seeking information [39,40].

In general, the overall results show that only one-third of the participants from the experimental group visited the database, a minority visited the website in detail, and that the evaluation of the website was not positive. It is important to mention that smokers had a lower intention to revisit the website again and these people are the target markets of this website. Visitors should benefit from the website, for example, in terms of increasing their knowledge. There may be several reasons why people do not visit a website in-depth: the layout, design, structure, and function of a website are essential elements that determine how a website will be perceived and used [41]. The evaluation of the website indicates important concepts of the website should be improved, such as understanding, relevance, trust, or enjoyment. Improving the website might motivate visitors to stay longer on the website, which in turn increases the chance that visitors receive information about tobacco additives. Furthermore, the website might be improved by changing the navigation into a clearer display, thereby avoiding hyperlinks within the text. Instead, a navigation path next to the main text may be provided. Given the fact that participants from this study did not visit pages with in-depth information about specific tobacco additives, it might be worth changing the content of the information into more general and less-specific information. This is because the majority of participants only looked at general information. More in-depth information about tobacco additives might be offered on the website and could also be labeled as “in-depth information” to help visitors to distinguish between broad and deep information. It might also be helpful to guide visitors through the website using options such as “what information are you looking for?” These recommendations must be examined in further studies.

### Limitations

There are some limitations to this study. Our participants were recruited via a panel and received a reward for participating. The information given on the website might have a greater impact on people who visited the website proactively. Yet, inclusion of visitors only could have jeopardized the generalizability of our findings to the overall Dutch population.

Our model of predicted website use had a very low explained variance (8%). This indicates that there must be other variables that we did not measured and are related to website use. It is conceivable that there are topic-related variables about tobacco additives that we did not assess, such as interest, or variables associated with Internet usage, including health literacy [42].

Furthermore, we only assessed which hyperlinks participants used; other measures of website engagement, such as time people spent online, might be of interest.

Another limitation of our study is that we cannot preclude the possibility that participants from the control group visited the original website between the baseline and the follow-up measurement. This is because the website could be found on the Web just as other websites about tobacco additives on the Web (eg, by means of a Google search). Finally, hyperlinks to topics other than tobacco additives on the website were deactivated, possibly resulting in bias to the observed website usage.

### Conclusion

With the tabakinfo website and disclosure of information about tobacco additives, the Dutch government fulfills their requirements to inform the general population [43]. Visiting the website did not influence knowledge, risk perception, attitudes toward tobacco additives, and smoking behavior. If website-based health-related information is to have an impact on concepts such as knowledge, risk perception, or attitude, it will be necessary to adapt the website more to the needs of the visitors. That could be achieved, for example, by making the information easily accessible on the home page, thus avoiding long browsing activities. This is because most of the participants only visited the first subwebsite. Furthermore, it may be desirable to make the website more attractive to those who are less educated and to smokers. It might also be also possible that the website was not encouraging participants to look up information in detail, but this notion needs to be evaluated through further studies. Additionally, as the needs of visitors may differ, tailoring the information to their needs is recommended. Indeed, further research is needed to gain a deeper insight into these needs and requirements.

## Abbreviations

PREP: potential reduced exposure product
RCT: randomized controlled trial

## Appendix

Multimedia Appendix 1 Questionnaire about tobacco additives.
