# Supplementary material for: Website Use and Effects of Online Information About Tobacco Additives Among the Dutch General Population: A Randomized Controlled Trial
Source: J Med Internet Res. 2017 Mar 14;19(3):e60. doi: 10.2196/jmir.6785 (PMC5373788; doi:10.2196/jmir.6785)
Supplement: Multimedia Appendix 1 [file jmir_v19i3e60_app1.pdf]

Appendix 1: Questionnaire about tobacco additives (TA)

Knowledge about TA

1. With the use of tobacco additives, inhaled smoke becomes milder and respiratory track irritations are limited
2. Tobacco with additives contains less carcinogenic substances in comparison to tobacco without additives
3. Sugar is an additive that is added to tobacco
4. Additives are only added to tobacco
5. Tobacco additives can increase addiction to tobacco
6. Cigarettes without additives are less harmful to health than cigarettes with additives
7. Toxic substances can be formed during the combustion of tobacco additives
8. Tobacco additives are added in order to improve taste
9. Additives protect against the harmful effects of smoking
10. Nicotine is added to tobacco in order to make it more attractive

Answer categories: right, wrong, I don't know (right 1,3,5,7,8,)

Risk perception regarding TAs

If I smoke tobacco with additives

1. … I have a high risk of getting cancer
2. … I become quickly addicted to tobacco
3. … I have a high risk for health problems

I have the feeling that…

4. … smoking tobacco with additives is harmful to my health
5. … if i smoke tobacco with additives I become more easily addicted to tobacco
6. … if i smoke tobacco with additives I have a high risk of developing health problems

Answers: totally agree, agree, neither agree nor disagree, disagree, strongly disagree)

Attitude regarding TAs

If I would smoke tobacco with additives…

1. …than I won't get a sore throat
2. … than I inhale less toxins
3. …then this would be less harmful to my health
4. …than this would be less addictive
5. … than I would have a good feeling
6. … than I would feel stressed
7. … than I could relax better
8. … than I would feel calm
9. …than this is harmful to my health
10. …than this is harmful to my environment
11. …than I inhale toxins
12. …it would have a distinguished taste
13. … than I would regret it
14. …than I would feel addicted

Answers: totally agree, agree, neither agree nor disagree, disagree, strongly disagree

Website evaluation

We would like to know how you evaluate the parts of the website you have seen.

Efficiency

1. I could easily search information on this website
2. I have quick access to information on this website
3. It took little effort to find information quickly on this website

Effectiveness

1. The website provided useful information about tobacco additives
2. The website provided relevant information about tobacco additives
3. The website helps me to give information about tobacco additives

Enjoyment

1. I liked my visit to the website
2. I thought my visit to the website was pleasant
3. I thought my visit to the website was nice

Active trust

1. I could act upon information provided on the website
2. I could use the information provided at this website in my daily life
3. I feel like I could use the information at this website if needed

Relevance

1. I found the information interesting
2. I found the information clear
3. The information was new to me

Understanding

1. The language used on the website is clear to me
2. I found the information easy to understand
3. I found many words on the website difficult to understand

Completeness

1. The website provides me with enough information
2. I found the information on the website accurate

Layout

1. I liked the layout of the website
2. I thought the website looked good

Recommending to others

1. It is likely that I would recommend the website to others.
2. I would recommend the website to others if they are looking for information about tobacco additives
3. I would recommend the website to others

Intention to revisit

1. It is likely that I will visit tabakinfo.nl again in the future
2. I have the intention to visit the website again if I am looking for information about tobacco additives
3. I would use the website again

Answers: totally agree, agree, neither agree nor disagree, disagree, strongly disagree
